# Potential Supportive Effects of AP Collagen Peptides on Skin Hydration in a Mouse Model of Oxazolone-Induced Atopic Dermatitis

**DOI:** 10.4014/jmb.2604.04022

**Published:** 2026-08-13

**Authors:** Su-Young Kim, A Yeon Park, Hyun Joo Lee, Eo Jin Kim, Ayeon Choi, Sun Young Choi, Beom Joon Kim

**Affiliations:** 1 Department of Dermatology, Chung-Ang University Hospital, Chung-Ang University College of Medicine, Seoul 06974, Republic of Korea; 2 Department of Medicine, Graduate School, Chung-Ang University, Seoul 06974, Republic of Korea; 3 Department of Dermatology, Chung-Ang University Gwang-Myeong Hospital, Chung-Ang University College of Medicine, Gyeonggi-do 14353, Republic of Korea; 4 Biomedical Research Institute, Chung-Ang University Gwangmyeong Hospital, Gyeonggi-do 14353, Republic of Korea

**Keywords:** Atopic dermatitis, Fish collagen peptides, Hydration, Adjunctive agent

## Abstract

Atopic dermatitis (AD) is a chronic and relapsing inflammatory skin disorder characterized by impaired skin barrier function and Th2-mediated immune responses. Collagen tripeptide (CTP), an amino acid–rich dietary supplement with low antigenicity, has been reported to improve skin hydration and barrier function. However, its therapeutic effects in AD, particularly under conditions of barrier dysfunction, have not yet been fully established. In this study, we evaluated the potential effects of AP collagen peptide (APCP), which contains CTP, on skin hydration and inflammatory responses, in an oxazolone (OXZ)-induced AD-like model using hairless mice. Skin hydration and transepidermal water loss (TEWL) were measured, while inflammatory responses were analyzed by histological evaluation and RT-qPCR. APCP-treated mice showed a tendency toward improvement in AD-like clinical symptoms and TEWL. In addition, APCP administration significantly increased skin hydration, accompanied by restoration of filaggrin expression and significantly reduced expression of TSLP, p-STAT3, and p-ERK. In contrast, mast cell infiltration, CD3^+^ and CD4^+^ T cell infiltration, and the expression of inflammatory cytokines, including IFN-γ, IL-4, IL-13, and IL-31, showed a tendency toward reduction but did not reach statistical significance. These findings suggest that APCP may not exert broad immunosuppressive effects, but may contribute to the alleviation of AD symptoms through improvements in skin hydration, supporting its potential as an adjunctive agent for AD.

## Introduction

Atopic dermatitis (AD) is a common long-term, relapsing inflammatory skin condition that primarily affects children, with a reported prevalence of up to 20%. The pathogenesis of AD involves a complex interplay of environmental, genetic, and immunological factors, resulting in hallmark symptoms such as pruritus, xerosis, and hyperkeratosis [1]. Recently, thymic stromal lymphopoietin (TSLP), an epithelial cell-derived cytokine, has been recognized as a key regulator in the initiation and progression of AD [2]. TSLP promotes type 2 immune responses, activates multiple inflammatory cell populations, and contributes to skin barrier dysfunction, in part by suppressing the expression of barrier-related proteins, including filaggrin [2, 3]. Reduced filaggrin expression is a hallmark feature of AD and impairs the skin's ability to retain moisture, thereby exacerbating xerosis and facilitating the penetration of allergens and irritants, and ultimately perpetuating cutaneous inflammation [4, 5]. Sensory nerve sensitization and pruritus in AD are mediated by interleukin (IL)-4, IL-13, and IL-31, which exacerbate inflammation and compromise skin barrier integrity [4, 6-10].

Systemic immunosuppressive agents, including corticosteroids, cyclosporine, and methotrexate, are commonly employed for AD management; however, long-term administration has been linked to serious side effects, including high blood pressure, liver toxicity, and kidney damage [6]. Therefore, adjunctive therapeutic strategies that can complement conventional treatments and improve clinical outcomes while minimizing treatment-related adverse effects are of increasing interest.

Recently, dietary supplements have increasingly been reported to help improve inflammatory skin disease such as rosacea [11], acne [12], and psoriasis [13, 14], gaining attention for their potential role in promoting skin health across various aspects [15, 16]. Collagen tripeptide (CTP), a highly purified derivative of collagen peptides characterized by its glycine-X-Y structure and produced via collagenase digestion, has emerged as a promising therapeutic candidate. CTP is associated with minimal antigenicity and a low risk of allergic reactions [17]. Oral administration of CTP has been shown to enhance fracture healing [18], ameliorate photoaging [19], and improve AD symptoms [20].

Among the newly developed CTP-based products, AP collagen peptide (APCP), a collagen tripeptide-rich hydrolysate extracted from the scales of golden threadfin bream (*Nemipterus virgatus*), contains over 15% CTP, including 3% glycine–proline–hydroxyproline [21]. It has demonstrated multiple dermatological benefits, including the inhibition of photoaging[22], induced hair growth [21, 23], mitigation of cortisol-induced aging [24], and enhancement of skin hydration in dry skin [25]. In this study, we investigated whether orally administered APCP could alleviate xerosis, a major clinical manifestation of AD, by improving skin hydration in an oxazolone-induced AD-like mouse model, and evaluated its potential as an adjunctive agent for the management of AD.

## Materials and Methods

### APCP Preparation

The APCP used in this study was prepared in powdered form by enzymatically hydrolyzing gelatin derived from the scales of golden threadfin bream (*Nemipterus virgatus*) using a specific collagenase, followed by concentration and drying (Amorepacific, Republic of Korea) [21]. These APCPs contained >15% CTP and 3% Gly–Pro–Hyp. All supplement manufacturing followed Good Manufacturing Practice guidelines in facilities certified under Hazard Analysis and Critical Control Points standards.

### Induction of AD-Like Lesions

AD-like lesions were generated in hairless mice through Oxazolone (Sigma, USA) administration as described by Man *et al*. [26] During the sensitization phase, mice were administered 10 μL of 5% OXZ in a 4:1 mixture of olive oil and acetone in the dorsal neck skin three weeks before starting the experiment. In the challenge phase (one week after sensitization), mice were administered 60 μL of 0.3% OXZ in the lower back skin every two days for two weeks. This was followed by daily oral administration of saline or APCP for 12 weeks, with concurrent treatment with 0.3% OXZ.

### Animals and Experimental Design

Six-week-old female hairless mice were obtained from Saeron Bio Inc. (Republic of Korea) and housed for one week to adapt to controlled environmental conditions (55 ± 10% relative humidity, 12-hour light/dark cycle, and 23 ± 2°C). The experiment protocol was approved by the Chung-Ang University Animal Laboratory Ethics Committee (Approval No. A2022035). We used the ARRIVE checklist [27] and randomly distributed the mice (n = 10 per group) into the normal group (Group 1; no treatment), the OXZ-only group (Group 2; treated with saline), the OXZ + APCP 200 group (Group 3; treated with APCP at 200 mg/kg in saline), and the OXZ + APCP 300 group (Group 4; treated with APCP at 300 mg/kg in saline).

### Measurement of Dermatitis Severity

Test animals were anesthetized with a mixture of Zoletil (40 mg/kg; 0.008 cc/10 g) and Rompun (5 mg/kg; 0.002 cc/10 g), which had been diluted tenfold in normal saline. Photographs of the dorsal skin were taken at close range using a digital single-lens reflex (DSLR) camera (Nikon, Japan) while the animals were under anesthesia. At the conclusion of the experiment, the severity of AD-like skin lesions on the dorsal area was evaluated based on the dermatitis scoring system described by Kang *et al*. [28]. Four clinical parameters—excoriation, scaling, edema, and erythema—were each rated on a 0 to 3 scale (0 = almost clear, 1 = mild, 2 = moderate, 3 = severe), with the total score ranging from 0 to 12.

### Skin Tissue Histological Analysis

On the last day of the study, the test animals were euthanized by CO_2_ asphyxiation, and skin samples were collected from the lesion sites for histological analysis. The biopsies were fixed in 10% neutral buffered formalin for 24 h and subsequently processed into paraffin-embedded sections with a thickness of 5 μm. These sections were affixed to POLYSINE slides (Thermo Fisher Scientific, USA), treated with xylene to remove wax, and sequentially dehydrated using an ethanol gradient. Hematoxylin and eosin (H&E) staining was performed to assess histological features, including skin thickness, while toluidine blue (TB) staining was used to identify mast cells in the skin. For immunohistochemical (IHC) analysis, additional sections were stained with primary antibodies targeting anti-CD3ε (99940S, Cell Signaling Technology, USA) and anti-CD4 (ab183685, Abcam, UK), anti-filaggrin (GeneTex, GTX37695), anti-TSLP (Abcam, ab188766), anti-p-ERK (CST, 4370S), anti-p-STAT3 (CST, 9145S). In parallel, the Vector Excel Amplified Polymer Kit, Peroxidase (anti-rabbit IgG; MP-7601-15, Vector Laboratories, USA) was used according to the manufacturer’s instructions. Following this, the slides were washed with phosphate-buffered saline containing Tween 20 (PBS-T) and treated with a high-sensitivity, two-component 3,3-diaminobenzidine (DAB) chromogenic substrate (Vector Laboratories). All stained slides were digitized with a Pannoramic MIDI slide scanner (3DHISTECH Ltd., Hungary), and the images were analyzed using CaseViewer software and ImageJ.

### Corneometer and Transepidermal Water Loss (TEWL)

Stratum corneum hydration levels (arbitrary units) and TEWL (g/m^2^/h) were assessed using a Corneometer^®^ CM 825 and a Tewameter (both from Courage Khazaka Electronic GmbH, Germany), respectively. All assessments were performed in controlled environmental conditions (room temperature, relative humidity: 50%–60%). Each parameter was recorded thrice (excluding the initial measurement), and the mean value was used for analysis.

### RNA Preparation and Reverse Transcription-Quantitative Polymerase Chain Reaction (RT-qPCR)

On the last day of the study, the test animals were euthanized by CO_2_ asphyxiation, and skin samples were collected from the lesion area for analysis. Total RNA was isolated using TRIzol reagent (Invitrogen, USA) following the manufacturer’s guidelines. Complementary DNA was synthesized using PrimeScript^TM^ RT Master Mix (Takara, Japan). Real-time PCR analysis was done using qPCR 2X PreMIX SYBR (Enzynomics, Republic of Korea) on a CFX-96 thermocycler (Bio-Rad, USA) using the following cycling conditions: 1) initial denaturation at 95°C for 10 min, followed by 30 cycles at 95°C for 10 sec, 60°C for 15 sec, and 72°C for 20 sec. Gene expression levels were determined using the 2^-ΔΔCt^ method and GAPDH as the reference gene. The primer sequences are listed in Table 1.

### Statistical Analyses

Results are presented as the mean ± standard error of the mean. Statistical analyses were performed using the Mann–Whitney test in GraphPad Prism 7.0 (GraphPad Software Inc., USA). A *p*-value of < 0.05 was considered statistically significant, and *, ***, and **** indicate *P* < 0.05, < 0.001, and < 0.0001, respectively.

## Results

### Oral Administration of APCP Alleviates AD-Like Symptoms

**Fig. 1:**
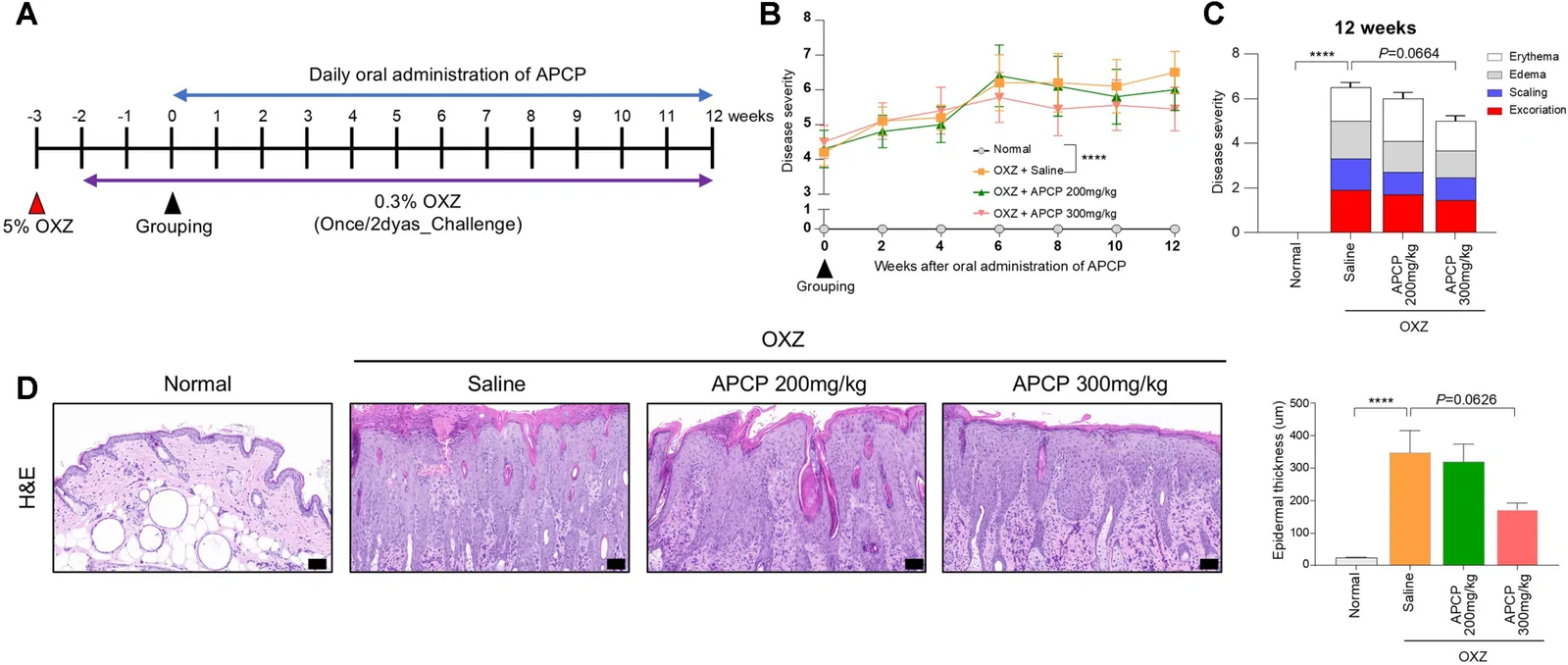
Oral APCP improves AD symptoms. (**A**) Experimental procedure in this study. (**B**) Changes in disease severity scoring over the entire experimental period by group (**B**), and (**C**) differences in disease severity scoring at week 12. (**D**) Histological analysis of epidermal thickness in dorsal skin lesions using H&E staining (Scale bar, 50 μm). The results are expressed as the mean ± SEM (n = 8~10 per group). Data were analyzed by two-way ANOVA followed by Bonferroni post hoc test (**B**) and Mann-Whitney test (C and D). *P < 0.05; ****P < 0.0001 compared with the OXZ+Saline group. *P* values shown above the brackets indicate comparisons between the connected groups.

To evaluate the potential effects of APCP on AD-like symptoms, 5% OXZ was topically applied once to the dorsal neck skin of mice at the -3 week time point. Subsequently, 0.3% OXZ was repeatedly applied to the lower back skin for two weeks to induce early AD-like lesions. Once lesion formation was confirmed, mice were grouped to ensure comparable disease severity across experimental groups. Thereafter, APCP was orally administered daily for 12 weeks, while 0.3% OXZ was continuously applied to the lower back skin to assess the potential efficacy of APCP in AD (Fig. 1A). Disease severity, as a clinical indicator after 12 weeks of oral APCP administration, showed a decreasing trend in the OXZ+APCP 300 mg/kg group compared to the OXZ+saline group (*P* = 0.0664) (Fig. 1B and 1C). The epidermal thickness in the OXZ+saline group was significantly increased, approximately 14.4-fold higher than that in the normal group. In contrast, the OXZ+APCP 300 mg/kg group showed a tendency toward reduced epidermal thickness, with approximately a 50% decrease compared with the OXZ+saline group (*P* = 0.0626) (Fig. 1D).

### APCP Improves Skin Hydration and Restores Filaggrin Expression Accompanied by Suppression of the TSLP/STAT3/ERK Pathway

**Fig. 2:**
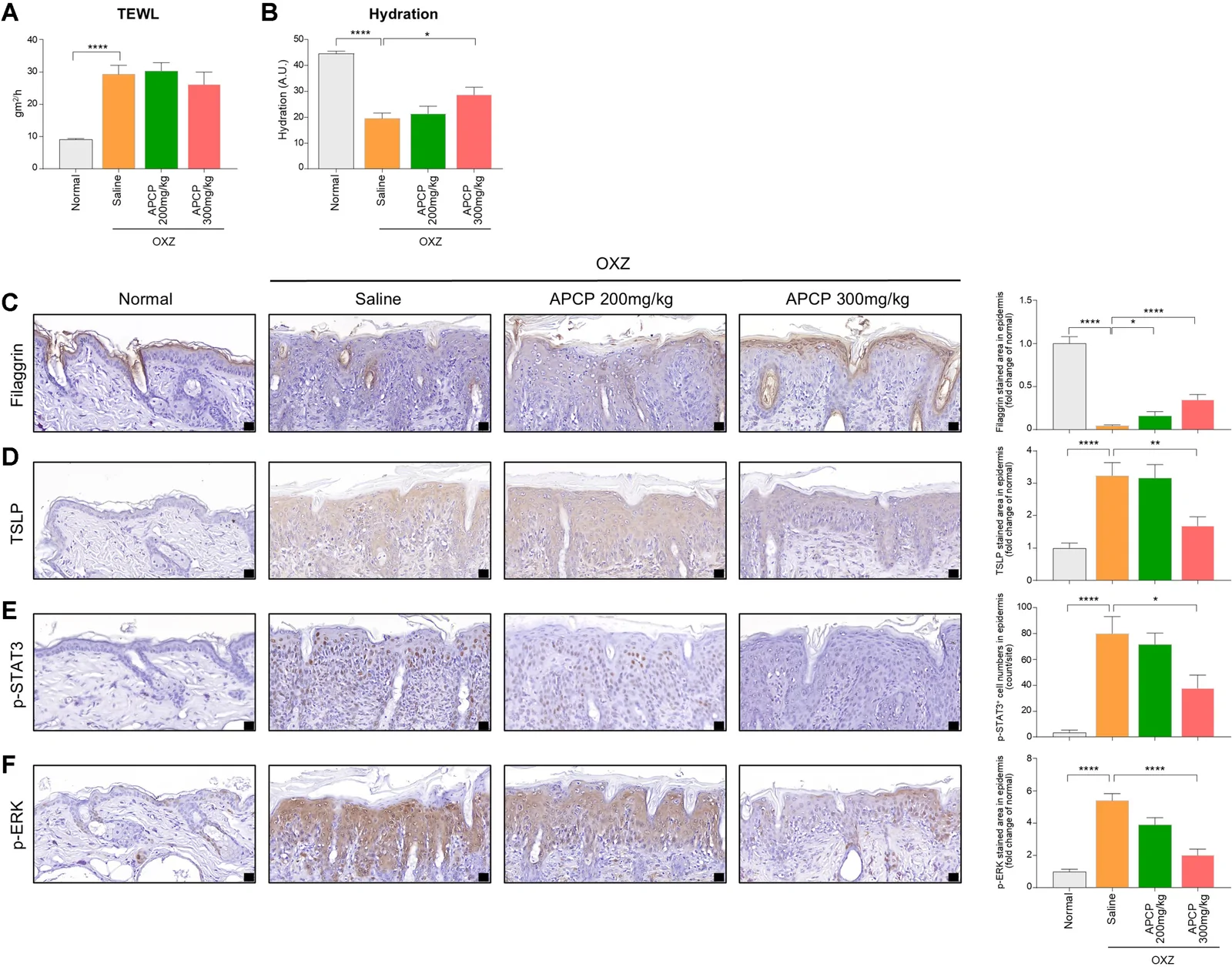
Oral APCP restores skin hydration and filaggrin expression with reduced TSLP, p-STAT3, and p-ERK expression in AD-like skin lesions. (**A−B**), At the end of the study, (**A**) TEWL and (**B**) hydration levels in AD-like mouse skin lesions were evaluated. (**C-F**) At the end of the experiment, skin lesions from AD-like mice were collected. Representative images of filaggrin (**C**), TSLP (**D**), p-STAT3 (**E**), and p-ERK (**F**) are shown (scale bar, 20 μm). DAB staining intensity was quantified using ImageJ, and the number of positive cells was counted. The results are expressed as the mean ± SEM (n = 8~10 per group). Data were analyzed by Mann-Whitney test (**A−F**). ***P < 0.001; ****P < 0.0001 compared with the OXZ+Saline group. *P* values shown above the brackets indicate comparisons between the connected groups.

To evaluate the effects of APCP on skin hydration in AD-like lesions, transepidermal water loss (TEWL) and skin hydration were assessed. Although TEWL was not significantly altered in the OXZ+APCP 300 mg/kg-treated group compared with the OXZ+saline group (Fig. 2A), skin hydration was significantly increased (Fig. 2B). In addition, the expression of filaggrin, a key structural component of the epidermal barrier, was significantly restored in the APCP-treated group compared with the OXZ+saline group (Fig. 2C). Consistent with these findings, the expression levels of TSLP (Fig. 2D), as well as p-STAT3 (Fig. 2E) and p-ERK (Fig. 2F), which are known to suppress filaggrin expression, were significantly decreased in OXZ+APCP 300 mg/kg-treated group.

### APCP Exhibits Limited Anti-Inflammatory Effects in AD-Like Lesions

**Fig. 3:**
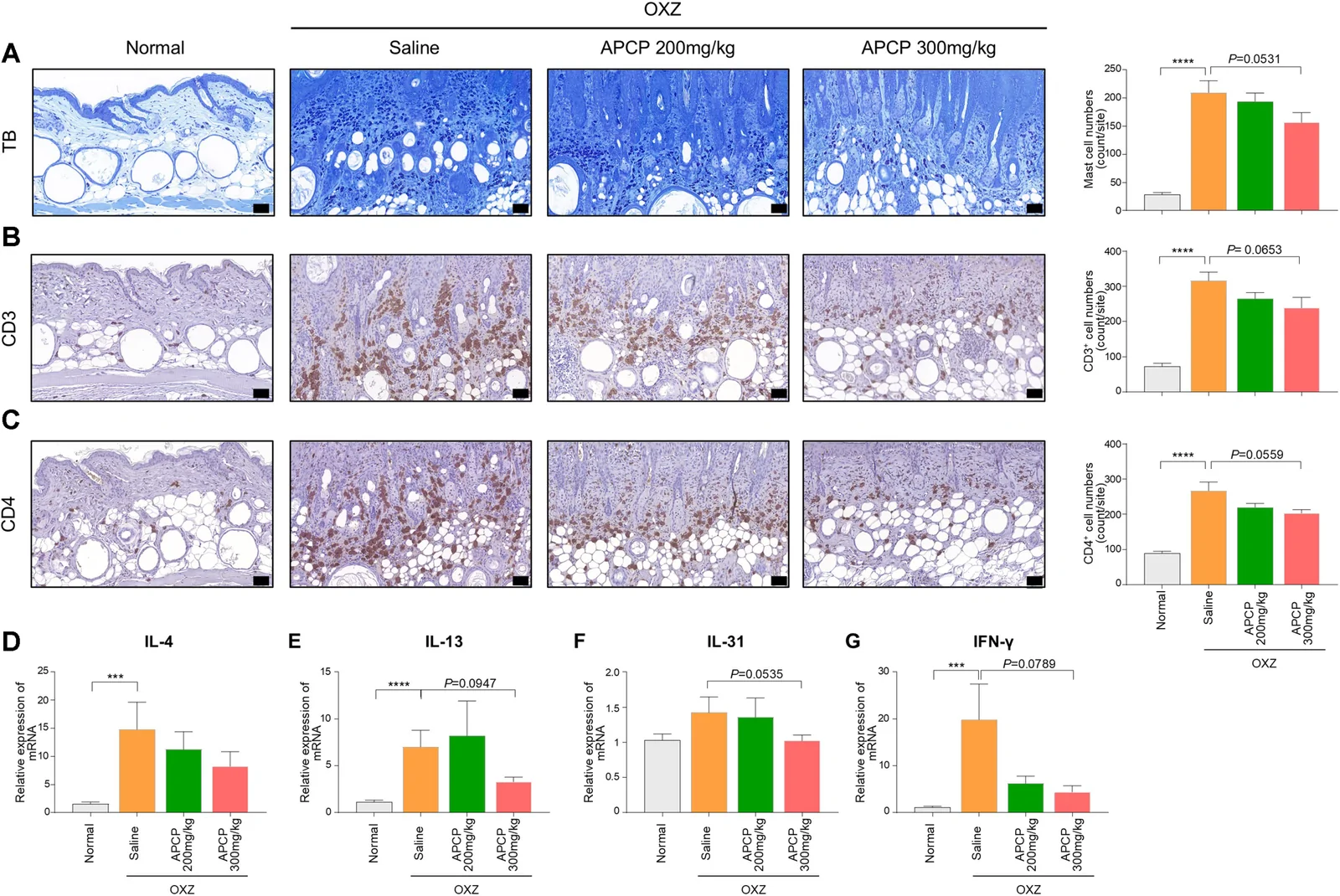
Oral APCP exhibits limited anti-inflammatory effects in AD-like skin lesions. Histological analysis of infiltration number of mast cells was measured using TB staining (**A**) (Scale bar, 50 μm). To assess T cell infiltration, the number of CD3^+^ (**B**) and CD4^+^ (**C**) (Scale bar, 50 μm) cells was counted based on DAB staining. The mRNA expression levels of IL-4 (**D**), IL-13 (**E**), IL-31 (**F**), and IFN-γ (**G**) in skin tissue were analyzed using RT-qPCR. The results are expressed as the mean ± SEM (n = 8~10 per group). Data were analyzed by Mann-Whitney test (D-G). ***P < 0.001; ****P < 0.0001 compared with the OXZ+Saline group. *P* values shown above the brackets indicate comparisons between the connected groups.

Inflammatory cell infiltration and cytokine expression were assessed to determine whether APCP modulates inflammatory responses in AD-like lesions. APCP treatment showed a tendency to reduce mast cell (*P* = 0.0531) (Fig. 3A), CD3^+^ (*P* = 0.0653) (Fig. 3B) and CD4^+^ T cell (*P* = 0.0559) (Fig. 3C) infiltration, although these changes were not statistically significant. Similarly, the expression levels of IL-4 (Fig. 3D), IL-13 (*P* = 0.0947) (Fig. 3E), IL-31 (*P* = 0.0535) (Fig. 3F), and IFN-γ (*P* = 0.0789) (Fig. 3G) tended to decrease in the APCP treated group compared with the OXZ+saline group. However, these differences did not reach statistical significance.

## Discussion

Various animal models have been developed to mimic the clinical and immunological features of AD, among which hapten-based models are the most widely used due to their high reproducibility and ease of application. Haptens are typically small molecules with a molecular weight of less than 1 kDa that cannot elicit an immune response on their own but become immunogenic when conjugated with carrier proteins [29]. One such hapten, OXZ, has been reported to induce contact dermatitis in mice, triggering Th2-like hypersensitivity reactions [26]. Repeated topical application of OXZ sensitizes the skin, and upon re-exposure, effector T cells are recruited, leading to inflammation primarily mediated by CD3^+^ and CD4^+^ T cells [30, 31]. This immune response closely resembles the Th2 cytokine-driven immune characteristics observed in human AD lesions. Therefore, in this study, the OXZ-induced dermatitis mouse model was employed to evaluate the therapeutic effects of APCP on AD.

APCP contains a substantial proportion of CTP, including Gly-Pro-Hyp, and is reported to exhibit superior absorption efficiency and bioavailability compared to other collagen peptides [32]. This peptide is rapidly absorbed and remains intact in the bloodstream, ensuring high stability. Notably, the Gly-Pro-Hyp component of APCP has been shown to facilitate the restoration of extracellular matrix-related gene expression and support the maintenance of skin homeostasis, thereby contributing to overall skin health [32].

To our knowledge, this is the first study to evaluate the effects of daily APCP administration over a 12-week period in a chronic mouse model of AD. No toxicity was observed throughout the oral administration period. Although APCP did not produce a statistically significant improvement in the clinical symptoms of AD and TEWL, it showed a tendency toward symptom improvement and significantly increased skin moisture content. These findings are consistent with previous clinical studies demonstrating that APCP supplementation significantly increased skin hydration content in healthy individuals, adults with dry skin, and even subjects with laser-induced skin injury, suggesting that its moisturizing effects may extend to conditions involving skin damage [25, 33, 34]. The moisturizing effect of APCP observed in the AD-like mouse model may be associated with the restoration of filaggrin expression through the downregulation of TSLP, p-STAT3, and p-ERK. TSLP and its downstream signaling molecules, p-STAT3 and p-ERK, have been reported to be involved in the suppression of filaggrin expression in keratinocytes [3]. Consistent with our findings, previous studies have also reported that CTP suppresses TSLP expression in normal human epidermal keratinocytes under AD-like inflammatory conditions *in vitro* [20]. Filaggrin, a skin barrier protein that is downregulated in patients with AD [35], serves as a major precursor of natural moisturizing factors in the stratum corneum [5]. Therefore, the APCP-induced reduction in TSLP, p-STAT3, and p-ERK may contribute to the restoration of filaggrin expression, thereby improving moisture retention capacity. Given that xerosis is a hallmark feature of AD, these findings suggest that APCP-mediated improvements in skin hydration may contribute to the alleviation of AD-associated symptoms.

In addition to its effects on skin hydration and filaggrin expression, we further investigated the potential anti-inflammatory effects of APCP in AD-like lesions by evaluating mast cell and T cell (CD3^+^ and CD4^+^ T cell) infiltration, as well as the expression of IL-4, IL-13, and IL-31. Although a general trend toward reduction was observed, these changes did not reach statistical significance. Taken together, these findings suggest that APCP may not exert broad anti-inflammatory effects or potent immunosuppressive activity in AD. However, APCP may provide adjunctive benefits in alleviating AD symptoms, primarily through improvements in skin hydration, with a possible contribution from the modulation of inflammation-related signaling pathways, including TSLP, p-STAT3, and p-ERK.

Several limitations of this study should be acknowledged. First, due to the inherent nature of animal studies, the sample size was relatively limited, which may have reduced the statistical power to detect biologically meaningful differences between groups. Second, increased within-group variability, driven in part by relatively high values observed in a small number of animals, may have contributed to the lack of statistical significance in several inflammatory parameters despite consistent trends toward improvement. Therefore, future studies involving larger cohorts and additional mechanistic investigations will be necessary to further clarify the potential benefits and mode of action of APCP.

In conclusion, APCP significantly improved skin hydration and restored filaggrin expression in an AD-like mouse model. Although its anti-inflammatory effects were limited, APCP may serve as a promising adjunctive agent for the management of skin dryness in AD.

## Figures and Tables

**Table 1:** RT-qPCR primer sequences.

Gene	Forward (5', 3')	Reverse (5'→3')
IL-4	GGTCTCAACCCCCAGCTAGT	GCCGATGATCTCTCTCAAGTGAT
IFN-γ	CACACTGCATCTTGGCTTTG	TCCACATCTATGCCACTTGAG
IL-13	CCTGGCTCTTGCTTGCCTT	GGTCTTGTGTGATGTTGCTCA
IL-31	TCAGCAGACGAATCAATACAGC	TCGCTCAACACTTTGACTTTCC
GAPDH	AGGTCGGTGTGAACGGATTTG	TGTAGACCATGTAGTTGAGGTCA
